# Alterations in the amino acid profile in patients with papillary thyroid carcinoma with and without Hashimoto’s thyroiditis

**DOI:** 10.3389/fendo.2023.1199291

**Published:** 2023-08-18

**Authors:** Andrzej Hellmann, Jacek Turyn, Agata Zwara, Justyna Korczynska, Aleksandra Taciak, Adriana Mika

**Affiliations:** ^1^ Department of General, Endocrine and Transplant Surgery, Faculty of Medicine, Medical University of Gdansk, Gdansk, Poland; ^2^ Department of Biochemistry, Faculty of Medicine, Medical University of Gdansk, Gdansk, Poland; ^3^ Department of Environmental Analysis, Faculty of Chemistry, University of Gdansk, Gdansk, Poland; ^4^ Department of Pharmaceutical Biochemistry, Faculty of Pharmacy, Medical University of Gdansk, Gdansk, Poland

**Keywords:** amino acids, Hashimoto’s thyroiditis, papillary thyroid cancer, serum, LC-MS

## Abstract

**Purpose:**

Amino acids (AAs) play important physiological roles in living cells. Some amino acid changes in blood are specific for autoimmune disorders, and some are specific for thyroid cancer. The aims of this study were to profile AA metabolites in the serum of patients with papillary thyroid carcinoma (PTC0) without Hashimoto’s thyroiditis (HT) and patients with PTC with HT (PTC1) and predict whether AA metabolites are associated with thyroid disease, thyroid hormone and thyroid autoantibodies.

**Methods:**

A total of 95 serum samples were collected, including 28 healthy controls (HCs), 28 PTC0 patients and 39 PTC1 patients. Serum samples were analyzed by high-performance liquid chromatography-triple stage quadrupole-mass spectrometry (HPLC-TSQ-MS), and twenty-one amino acids (AAs) were detected.

**Results:**

The serum concentration of glutamic acid was significantly elevated in PTC1 patients compared with PTC0 patients. Lysine was the second amino acid that differentiated these two groups of PTC patients. In addition, the serum concentrations of glycine, alanine and tyrosine were significantly reduced in both PTC patient groups compared to the HC group. These AAs were also correlated with thyroid hormones and antibodies. Five amino acid markers, namely, glycine, tyrosine, glutamic acid, glutamine and arginine, separated/distinguished PTC0 patients from healthy subjects, and eight AA markers, the same AAs as above without arginine but with alanine, leucine, valine and histidine, separated/distinguished PTC1 patients from healthy subjects based on ROC analysis.

**Conclusion:**

Compared with the HCs, changes in AAs in PTC0 and PTC1 patients showed similar patterns, suggesting the possibility of a common pathophysiological basis, which confirms preliminary research that PTC is significantly associated with pathologically confirmed HT. We found two AAs, lysine and alanine, that can perform diagnostic functions in distinguishing PTC1 from PTC0.

## 1 Introduction

Thyroid cancer (TC) is responsible for over 1% of neoplasms diagnosed every year in the general population. In Europe, there are approximately 3,500 new cases each year (1). Females are involved 3–5 times more often than men. The incidence of TC has increased rapidly in the last several years. In terms of short-term prognosis, it may become the second most common malignant neoplasm in women. Papillary thyroid cancer (PTC) accounts for more than 90% of all thyroid neoplasms (2) with a global incidence of 586,000 cases (3). Although PTC is related to an indolent disease course and has a favorable prognosis, it is a major challenge to stratify patients by risk of mortality or recurrence. Currently, clinicopathologic features associated with an unfavorable prognosis include older age, large tumor size, extrathyroidal extension (ETE), lymph node metastasis (LNM) and distant metastasis. Individuals with those features require more aggressive treatment (4, 5). On the other hand, low-intensity treatment or even active surveillance may be sufficient for patients who do not have these risk factors. Although some recent studies have indicated that Hashimoto’s thyroiditis (HT) (6) may be a tumor-promoting factor, HT-related issues have barely been mentioned in current TC treatment guidelines. The link between chronic inflammation and cancer is well described (7); however, it is generally associated with indolent potential in the GI tract, liver, and skin (8). There are several theories to explain the potential relationship; for example, misbehaved follicular epithelial regeneration following chronic inflammatory damage (9) or enhanced TSH stimulus together with additional inflammatory cytokines act as potential activators of aberrant cell proliferation (10). However, the exact molecular pathomechanism remains unclear. Autoimmune thyroid diseases, including Hashimoto’s thyroiditis (HT), are T-cell‐mediated organ‐specific autoimmune diseases, and the annual incidence of Hashimoto thyroiditis worldwide is estimated to be 0.3-1.5 cases per 1000 persons (11). HT, affects women 7-10 times more often than men (12). Prevalence increases with age, especially in patients diagnosed with other autoimmune conditions. There are various signs and symptoms of HT mainly due to hypothyroidism, including cool and dry skin, coarse hair, loss of body hair, and hyperlipidaemia (13). Chronic HT-induced inflammation may be associated with an increased risk of thyroid cancer. A recent meta-analysis reported that the rate of HT in PTC patients ranged between 4.75 and 38.4%, whereas the rate of PTC in HT patients ranged between 0.12 and 64.3% (14). The immune responses against PTC and HT are different. In PTC, the immune system is more silent and allows tumor progression, while in HT, the reaction is aggressive, destroying the proper functioning of the gland. According to some authors, HT is associated with a better prognosis due to an enhanced immune response and better control of tumor progression (15). However, the role of HT in PTC seems ambiguous and should be elucidated. It has been proven that HT plays a role in protein metabolism (16). As the basic building blocks of peptides and proteins, amino acids have a variety of physiological functions. Serum levels of polyamine metabolites were found to differ between patients with autoimmune thyroid disease and healthy controls (17). Hypothyroid status is related to lower alanine, aspartate, and glutamate concentrations. It is mainly caused by decreased whole-body proteolysis and maldigestion (18). Amino acids are the primary units of proteins and are involved in multiple physiological and pathophysiological processes (19). According to many publications, amino acids (AAs) may play a critical role in cancer cell metabolism. In contrast to hypothyroidism status, it is known that in cancer, especially in the early stages, the amino acid turnover rate is increased because of hypermetabolism. Glutamine, as a nitrogen and carbon source, is involved in the metabolic reprogramming in cancer and plays a pivotal role in the growth and proliferation of cancer cells (18, 20–22). Most thyroid cancer studies have presented increased concentrations of glutamine and glutamate in tumor samples (23–25), as well as in serum (25–27). High glutamine uptake is related with upregulated glutaminase in several tumor models (28–30). Glutaminase initiates glutaminolysis by converting glutamine to glutamate. This pathway is involved in the maintenance of the TCA cycle and synthesis of non-essential amino acids, nucleotides and fatty acids (11) as well as in cell signaling (29, 30). Also, alteration of arginine metabolism is characteristic for cancer metabolism. It is necessary for growth of cancer cells, but paradoxically, arginine is important for immune surveillance (31). Alanine also is desired amino acid in most of the perturbed pathways (25). Glycine and serine provide crucial substrates for the synthesis of nucleic acids, proteins and lipids, which are essential for cancer cell growth (32). High levels of glycine have been observed in cancer thyroid tissues (24, 33) and in malignant nodules (34) compared to samples from healthy subjects. Also, other amino acid are necessary for maintenance of cellular redox homoeostasis (35). Some derivatives produced from AAs may support cancer growth, but tryptophan induces immunosuppression by weakening the ability of dendritic cells and T cells to target and eliminate cancer cells (36). Our aim was the examination of AA profile disorders in PTC1 and PTC0 and comparison of changes in AA concentrations in these 2 pathologies.

## 2 Materials and methods

### 2.1 Patients

The present study was approved by the Independent Bioethics Committee for Scientific Research at the Medical University of Gdansk under number NKBBN/62/2021. The study was performed in agreement with the Declaration of Helsinki of the World Medical Association. Female patients who underwent thyroidectomy or lobectomy for PTC at the Thyroid Cancer Center of the Medical University of Gdansk from January 1, 2021, to March 31, 2022, were included in the study. The study groups consisted of 28 PTC0 (mean age 42.3 ± 13.7 years) and 39 PTC1 patients (mean age 42.0 ± 14.1 years). The controls were healthy participants (43.6 ± 8.87 years). An extensive medical history was taken from the control group regarding various ailments (hypertension, chronic kidney disease, heart failure, ischemic heart diseases, cerebrovascular, dyslipidemia, diabetes mellitus, type 2 diabetes mellitus, thyroid diseases) and taken drugs. The control group consisted of women without the above diseases. Written informed consent was obtained from all participants. Data such as age, sex, preoperative serum autoantibody levels, tumor characteristics, and treatment modalities were obtained from the medical records. Standard pathologic diagnoses were based on World Health Organization criteria (37). Routine laboratory parameters were determined at the Central Clinical Laboratory at the Medical University of Gdansk, the results of which are collected in **Table 1**. Only patients with confirmed PTC by histopathology were included in the study. Coexistent HT was determined by elevated anti-thyroglobulin antibodies (TgAbs) and thyroid peroxidase antibody (TPOAb) and postoperative sectioning and examination of paraffin-embedded thyroid tissue specimens; a positive result was defined as the presence of diffuse lymphocytic and plasma cell infiltrate, oxyphilic cells, formation of lymphoid follicles, and reactive germinal centers. Only women participated in this study, and we ruled out other autoimmune thyroid diseases, such as Graves’ disease, through the determination of the levels of thyrotropin receptor autoantibodies (TSHR-Abs). Blood samples were collected in the morning from all study subjects, and before thyroidectomy from PTC patients. After the blood was centrifuged, the serum samples obtained were stored in aliquots at -80 °C until assayed.

**Table 1:** Selected biochemical and anthropometric characteristics in the study groups.

	HC	PTC0	PTC1	HC vs PTC0	HC vs PTC1	PTC0 vs PTC1
Age (year)	43.6 ± 8.87	42.3 ± 13.7	42.0 ± 14.1	NS	NS	NS
BMI (kg/m2)	25.4 ± 7.31	26.5 ± 4.47	25.1 ± 4.45	NS	NS	NS
TG (mg/dL)	98.5 ± 44.6	87.3 ± 32.6	81.6 ± 32.3	NS	NS	NS
HDL (mg/dL)	68.8 ± 18.0	62.0 ± 12.8	59.8 ± 12.5	NS	NS	NS
LDL (mg/dL)	101 ± 27.4	112 ± 35.4	109 ± 33.6	NS	NS	NS
TC (mg/dL)	160 ± 50.9	199 ± 42.2	188 ± 42.0	*0.014	NS	NS
CRP (mg/L)	0.77 ± 0.54	0.84 ± 0.52	2.77 ± 1.99	NS	<0.001	<0.001
Glucose (mg/dL)	ND	93.9 ± 27.0	95.3 ± 20.3	ND	NS	NS
HBA1C (%)	ND	5.37 ± 0.46	5.29 ± 0.34	ND	NS	NS
Insulin (uU/mL)	ND	9.14 ± 5.86	8.24 ± 6.05	ND	NS	NS
Albumin (g/L)	ND	42.0 ± 2.48	40.7 ± 3.46	ND	NS	NS
Creatinine (mg/dL)	ND	0.68 ± 0.11	0.69 ± 0.13	ND	NS	NS
1,25-(OH)_2_D (pg/mL)	ND	54.8 ± 15.0	51.3 ± 17.1	ND	NS	NS
TSH (uU/mL)	ND	1.14 ± 0.67	1.19 ± 0.80	ND	NS	NS
fT3 (pmol/L)	ND	4.30 ± 0.48	4.30 ± 1.06	ND	NS	NS
fT4 (pmol/L)	ND	12.3 ± 1.83	12.8 ± 3.03	ND	NS	NS
^a^Anty-TSHr (IU/l)	ND	<0.20	<0.20	ND	NS	NS
^b^Anty-TPO (IU/mL)	ND	<3.00	449 ± 532	ND	ND	**<0.001
^c^Anty-TG (IU/mL)	ND	<3.00	133 ± 471	ND	ND	**<0.001

p from one-way analysis of variance followed by the all-pairwise comparisons Holm−Sidak method, * p from nonparametric Kruskal−Wallis one-way analysis of variance followed by the all-pairwise comparisons Dunn’s method for ranks. ** <0.001 - comparison between the two PTC study groups was evaluated by the Mann–Whitney rank sum test for nonparametric data. ^a <^0.2 IU/l – reference value for TSHr-Ab, ^b^<34 IU/ml - reference value for TPO-Ab, ^c ≤^115 IU/ml - reference value for TG-Ab. ND – not determined, NS – not significant.

Healthy control (HC), patients with PTC without Hashimoto thyroiditis (PTC0) and patients with Hashimoto thyroiditis (PTC1). Values are mean ± SD.

### 2.2 Amino acid analysis

Concentrations of amino acids were determined by liquid chromatography/mass spectrometry (LC/MS) according to the procedure described previously (38). Briefly, internal standards (a mixture of amino acids labelled with stable isotopomers C-13 and N-15, Sigma−Aldrich) were added to 0.025 ml of serum. The sample was then deproteinized by the addition of 0.1 ml acetonitrile, incubated for 15 minutes on ice and centrifuged at 12,000 x g for 15 minutes at 4°C. The collected supernatant was freeze-dried and then dissolved in 25 µl of water. Samples were analyzed by ion-pair reversed-phase high-performance liquid chromatography coupled with mass spectrometric detection. Chromatographic separation was performed using a 2.5 μm Synergy Hydro-RP 50 x 2.0 mm column. The mobile phase was delivered at a rate of 0.2 mL/min in a gradient from 0% to 60% acetonitrile over 12 minutes. A mass detector (TSQ Vantage, Thermo, USA) with a heated electrospray ion source (HESI-2) was operated in MS2 positive mode for amino acid detection. The electrospray cone voltage was set at 4.5 kV, and the heated capillary temperature was 275°C. The sheath gas flow was set at 35 arbitrary units. Individual amino acids were identified and confirmed by the similarity of molecular masses, chromatographic retention time and fragmentation pattern.

### 2.3 Data analysis

The data analysis was performed in SigmaPlot 14.5 (Systat Software Inc., San Jose, CA, USA). All values are presented as the mean ± standard deviation (SD). The P value was considered significant at < 0.05. Comparisons among the three study groups were carried out with the one-way analysis of variance (ANOVA) followed by the all-pairwise comparison Holm−Sidak method. Nonparametric data were subjected to the Kruskal−Wallis one-way analysis of variance followed by the all-pairwise comparison Dunn’s method for ranks. Comparison between the two PTC study groups was evaluated by the Mann–Whitney rank sum test for nonparametric data. Correlations between pairs of variables were determined by linear regression analysis.

ROC analysis was carried out in MetaboAnalyst 5.0v (39) to evaluate the area under the curve (AUC) to compare the predictive ability of significant metabolites between the tested groups. The linear SVM algorithm was used to build the ROC curve. To understand if it is possible to increase the predictive power, the single ROC curve was built for both comparisons, HC with PTC0 and HC with PTC1, using only the metabolites with a p-value <0.01. ROC curve analyses for combinatorial AAs, the 10-fold Coss Validation was used to generate a logistic regression model and calculate the performance. MetaboAnalyst 5.0v uses the MetaboAnalyst R package with metabolomic data analysis, visualization, and functional interpretation. The raw data were subjected to normalization to the total area and autoscaled.

MetPA software (39) was used to carry out an analysis of serum metabolic pathways for the identified metabolites. Metabolome analysis identified all matched pathways based on p values determined during pathway enrichment analysis and pathway impact values determined by pathway topology analysis. The raw data were subjected to normalization to the total area and autoscaled. The pathway-associated metabolite set was the chosen metabolite library, and all compounds in this library were used. Pathways with a p value <0.05 were significantly altered in serum samples.

## 3 Results

Common biochemical parameters obtained from whole blood are presented in **Table 1**. PTC1 patents had elevated C-reactive protein compared with HC and PTC0 patients (although the values were within the reference range). PTC0 patients had significantly elevated concentrations of total cholesterol compared with HCs. Among the other parameters, significant differences were not observed. PTC0 patients differed statistically from PTC1 patients in thyroid peroxidase and thyroglobulin antibody levels.

### 3.1 Differences in serum AA concentrations in PTC patients with and without Hashimoto’s disease and healthy controls

**Figure 1:**
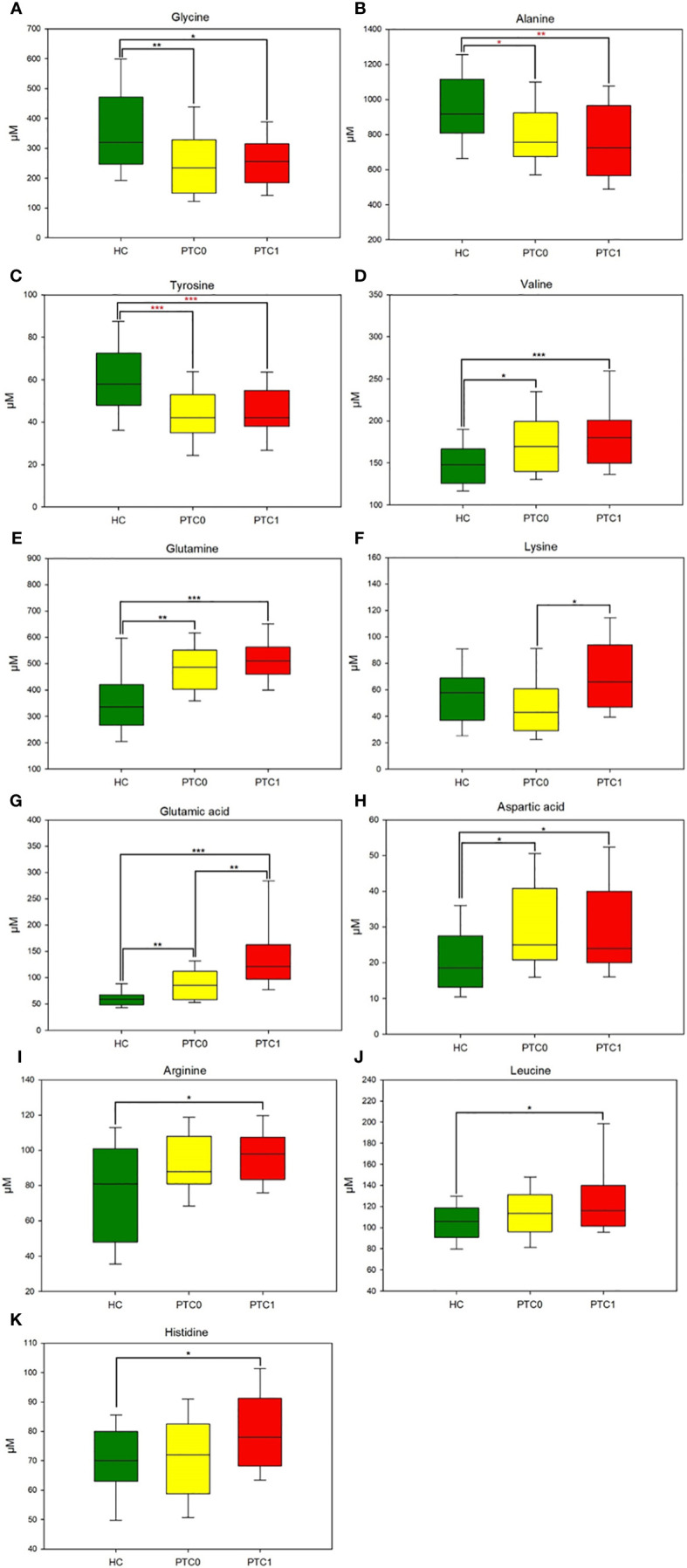
Boxplots of the 11 most significant metabolites (p < 0.05) in the analysis of variance results comparing the three groups (PTC0, yellow boxes; PTC1, red boxes; and healthy controls, green boxes). **(A)** glycine, **(B)** alanine, **(C)** tyrosine, **(D)** valine, **(E)** glutamine, **(F)** lysine, **(G)** glutamic acid, **(H)** aspartic acid, **(I)** arginine, **(J)** leucine, **(K)** histidine. The x-axis shows the specific metabolite, and the y-axis is the normalized peak intensity. HC, healthy control; PTC0, papillary thyroid carcinoma without Hashimoto; PTC1, papillary thyroid carcinoma with Hashimoto. Values are means ± SDs. (***p<0.001, **p<0.01, *p<0.05 one-way analysis of variance followed by the all-pairwise comparisons Holm−Sidak method; ***p<0.001, **p<0.01, *p<0.05 from nonparametric Kruskal−Wallis one-way analysis of variance followed by the all-pairwise comparisons Dunn’s method for ranks).

One-way analysis of variance was used to compare individual amino acids between study groups, and the significantly different AAs among these three groups were defined (**Table S1**). Concentration of some of these AAs decreased in the serum of both PTC0 and PTC1 patients due to the increased metabolic rate, which is typical of cancer. Glycine, alanine and tyrosine were reduced in both PTC groups compared with the HC group (**Figures 1A-C**). However, the values for glycine and valine (**Figure 1D**) were comparable for PTC0 and PTC1 patients, while the concentration of alanine showed a declining trend in the PTC1 group compared to that in the PTC0 group. In the PTC1 group, glutamate and lysine were significantly elevated in patients’ serum compared to the PTC0 group, and there were only two AAs that separated/distinguished these two groups of patients with PTC (**Figures 1E, F** and **Table S1**). All PTC patients had elevated levels of glutamic acid, aspartic acid, glutamine and valine compared to the healthy controls, and glutamic acid was noted to be almost two times higher in PTC1 patients than in PTC0 patients (**Figures 1D, E, G, H**). Significantly elevated concentrations of arginine, leucine and histidine were observed only in the PTC1 group compared with the healthy control group (**Figures 1I-K**). The increase in histidine was slight. In the PTC0 group, only leucine and arginine showed an upwards trend (**Table S1**).

### 3.2 Diagnostic potential of serum AA concentrations in PTC patients

ROC curve analysis of each box plot was used to evaluate the diagnostic ability of the discriminating metabolites as screening biomarkers in patients with PTC0 and PTC1. The ROC curve summarizes the specificity and sensitivity (the x-axis and y-axis, respectively) of a single feature to accurately classify data, which can then be used to compare the overall accuracy of different biomarkers.

**Figure 2:**
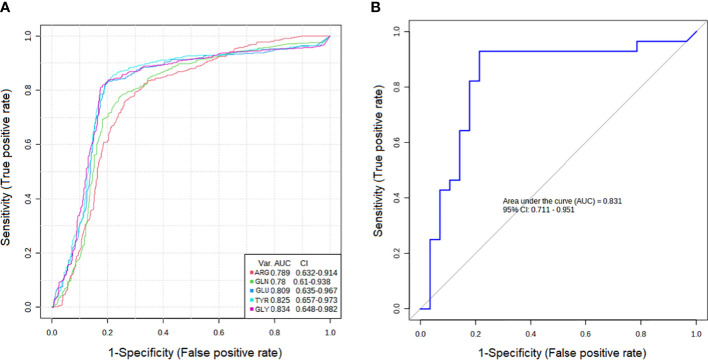
Receiver operating characteristic curve (ROC curve) analyses of the ability of 5 AAs **(A)** and a combinatorial AAs **(B)** to predict PTC0 vs. HC.

**Figure 3:**
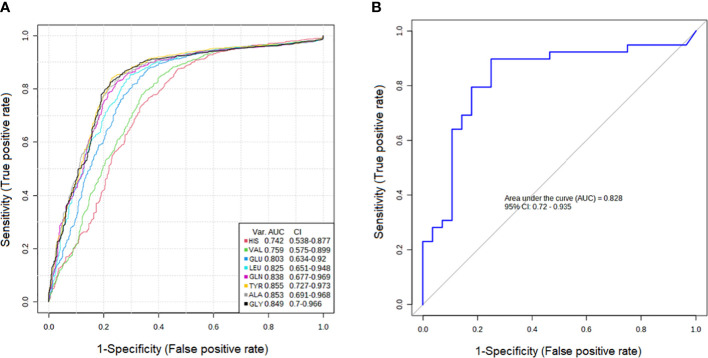
Receiver operating characteristic curve (ROC curve) analyses of the ability of 8 AAs **(A)** and a combinatorial AAs **(B)** to predict PTC1 vs. HC.

The results showed that the AUCs of five metabolites in the PTC0 vs. healthy group (**Figure 2A**) were larger than 0.780, and the AUCs of eight metabolites in the PTC1 vs. healthy group were larger than 0.742 (**Figure 3A**). Specific changes for PTC0 were found in arginine with an AUC of 0.789, and specific changes for PTC1 were found in alanine with an AUC of 0.853, leucine with an AUC 0.825, valine with an AUC 0.759 and histidine with an AUC of 0.0.742. The remaining AAs had different AUC values between PTC0 and PTC1. The highest AUC values noted was glycine in PTC0 and PTC1 (0.834 and 0.849, respectively) (**Figures 2**, **3**). As shown in **Figures 2B**, **3B**, the ROC curve for the predictive power of combined index to distinguish PTC0 from HC and PTC1 from HC was plotted. The AUC was 0.831 and 0.828, respectively (**Figures 2B**, **3B**).

### 3.3 Analysis of correlations between serum AA and concentrations and other selected blood parameters

The next step was the analysis of correlations between patient serum parameters of thyroid function and serum AAs in the research PTC groups (**Table 2**). Alanine was negatively correlated with free thyroxine (fT4). Arginine was positively correlated with fT4 (0.407, p<0.05) and leucine with TSH (-0.428, p<0.05). Only proline correlated with free triiodothyronine (fT3) (-0.426, p<0.02). There was only one strong negative correlation of histidine with C-reactive protein (-0.626, p<0.001).

**Table 2:** Correlation coefficients between the selected blood parameters and amino acid concentrations (µM) in serum samples from patients with PTC without Hashimoto thyroiditis (PTC0) (Pearson correlation coefficient).

	Asp	Asn	Gly	Gln	Glu	Ser	Bet	Thr	Ala	Pro	Cre	Val	Met	Tyr	His	Ile	Lys	Leu	Arg	Phe	Trp
CRP	0.220	**-0.385**	-0.091	-0.264	-0.130	-0.303	-0.247	-0.322	-0.109	-0.165	-0.037	-0.159	0.196	0.112	*-0.626*	0.006	-0.172	-0.004	-0.180	0.177	**-0.411**
TSH	-0.271	0.074	-0.108	-0.148	-0.126	0.125	-0.256	-0.036	-0.022	-0.334	**0.386**	-0.203	-0.264	-0.332	0.082	-0.095	-0.006	**-0.428**	-0.297	-0.012	0.020
fT3	-0.300	0.151	0.050	-0.171	0.055	-0.117	-0.139	-0.017	-0.221	**-0.426**	0.020	-0.270	-0.359	-0.104	0.294	-0.154	0.132	0.081	0.209	0.138	0.062
fT4	-0.208	-0.132	0.189	-0.236	0.212	-0.103	-0.117	-0.340	**-0.397**	-0.245	-0.111	0.000	0.100	0.114	-0.290	0.024	0.235	0.236	**0.407**	-0.154	-0.087
anty-TPO	–	–	–	–	–	–	–	–	–	–	–	–	–	–	–	–	–	–	–	–	–
anty-TG	–	–	–	–	–	–	–	–	–	–	–	–	–	–	–	–	–	–	–	–	–

Asp – aspartic acid, Asn – asparagine, Gly – glycine, Gln – glutamine, Glu – glutamic acid, Ser – serine, Bet – betaine, Thr – threonine, Ala – alanine, Pro – proline, Cre – creatinine, Val – valine, Met – methionine, Tyr - tyrosine, His - histidine, Ile – isoleucine, Lys – lysine, Leu – leucine, Arg – arginine, Phe – phenylalanine, Trp – tryptophane.

**p<0.05**; *p<0.001*.

Significantly more relationships and stronger correlations were observed in the PTC1 group, similar to the ANOVA and ROC analysis (**Table 3**). Among them were AA correlations with thyroid hormones. The PTC1 entity affects a greater number of correlations. Tyrosine, which was reduced in both the PTC0 and PTC1 groups, was positively correlated with fT3 (0.469, p<0.01) and fT4 (0.460, p<0.01). Lysine positively correlated with thyroglobulin antibodies (TG-Abs) (0.434, p<0.01) and was one of two AAs that were different between PTC0 and PTC1 (**Table S1**). In turn, alanine, which was also reduced in PTC1, was strongly negatively correlated with thyroid peroxidase antibodies (TPO-Abs) (-0.567, p<0.001). Another strong correlation was the positive correlation of glutamic acid with thyroid-stimulating hormone (TSH) (0.530, p<0.001).

**Table 3:** Correlation coefficients between the selected blood parameters and amino acid concentrations (µM) in serum samples from patients with PTC with Hashimoto thyroiditis (PTC1) (Pearson correlation coefficient).

	Asp	Asn	Gly	Gln	Glu	Ser	Bet	Thr	Ala	Pro	Cre	Val	Met	Tyr	His	Ile	Lys	Leu	Arg	Phe	Trp
CRP	-0.309	-0.212	-0.124	-0.294	-0.285	0.149	-0.262	-0.006	-0.022	-0.067	-0.085	0.137	-0.091	-0.137	0.141	0.174	-0.165	0.266	0.210	0.041	0.261
TSH	**0.356**	-0.095	0.105	0.109	*0.530*	-0.051	-0.010	-0.228	-0.097	**0.362**	0.088	0.046	0.070	-0.005	0.007	0.126	0.243	0.198	-0.026	-0.002	-0.063
fT3	-0.217	**0.377**	-0.117	0.030	-0.301	0.199	0.027	0.288	0.290	0.202	0.223	0.104	-0.077	**0.469**	0.077	-0.021	-0.237	-0.013	-0.310	0.112	0.085
fT4	-0.308	0.247	**-0.352**	-0.137	-0.171	0.177	-0.100	0.180	0.154	0.258	0.184	-0.087	0.013	**0.460**	0.105	-0.009	-0.213	0.033	-0.277	0.003	0.159
anty-TPO	0.103	-0.182	-0.278	-0.153	0.268	0.083	-0.244	0.013	*-0.567*	-0.169	-0.110	-0.184	-0.219	-0.150	-0.038	-0.092	0.040	-0.112	-0.154	-0.250	-0.003
anty-TG	0.215	-0.088	-0.016	-0.026	0.001	-0.195	-0.173	0.078	-0.272	-0.144	-0.318	-0.238	-0.198	-0.062	0.096	-0.086	**0.434**	-0.064	-0.117	0.023	0.018

Asp – aspartic acid, Asn – asparagine, Gly – glycine, Gln – glutamine, Glu – glutamic acid, Ser – serine, Bet – betaine, Thr – threonine, Ala – alanine, Pro – proline, Cre – creatinine, Val – valine, Met – methionine, Tyr - tyrosine, His - histidine, Ile – isoleucine, Lys – lysine, Leu – leucine, Arg – arginine, Phe – phenylalanine, Trp – tryptophane.

**p<0.05**; *p<0.001*.

### 3.4 Metabolic pathway analysis of the serum AA profiles in PTC0 and PTC1 patients

**Figure 4:**
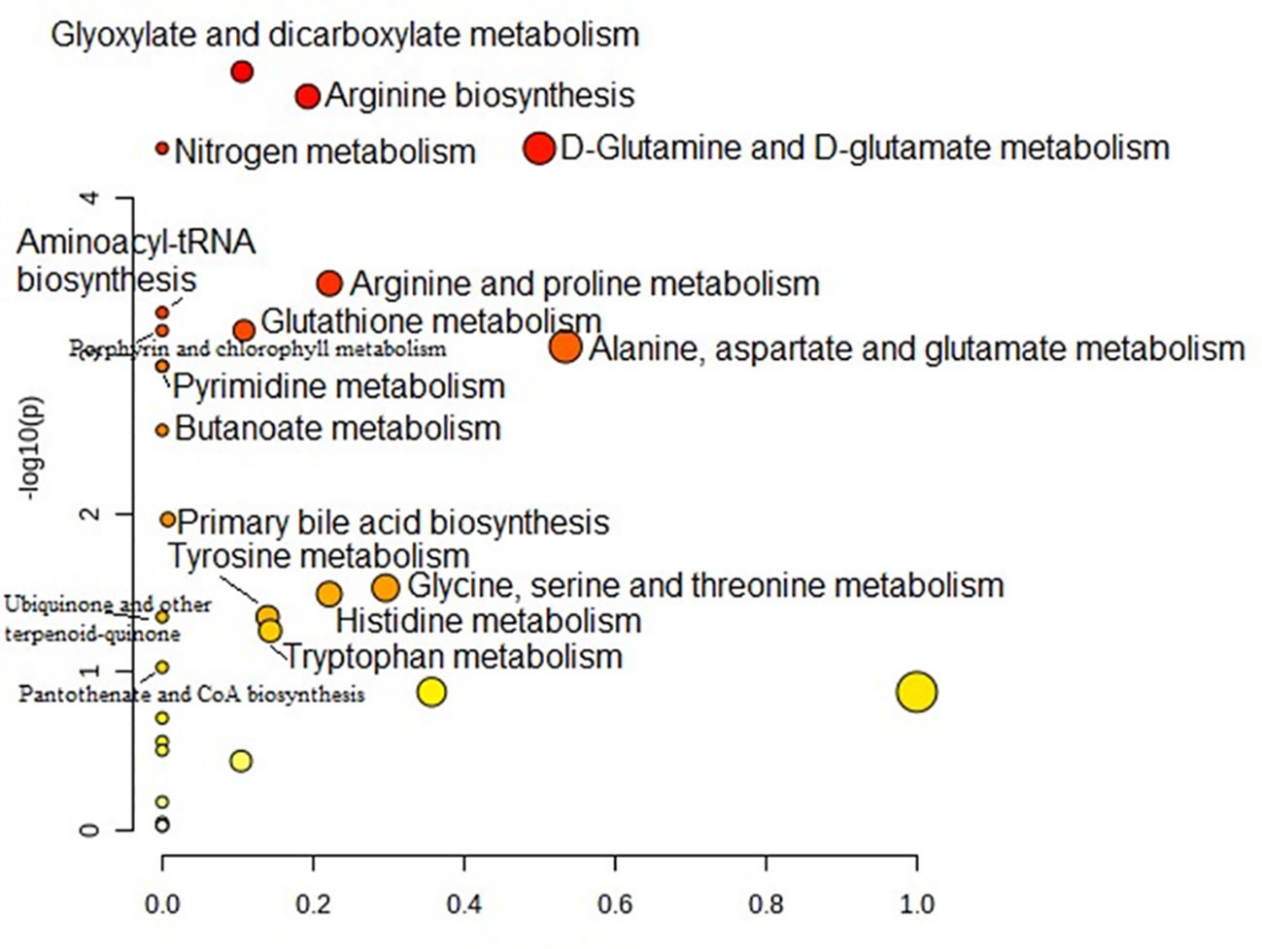
Pathway analysis of serum amino acid profiles of the papillary thyroid carcinoma without Hashimoto group compared to the control group. Pathway impact values are plotted against the X-axis, and -log (P) values are plotted against the Y-axis. For visual clarification, the pathway importance and the statistical significance are proportional to the node radius and colour, respectively. FDR p is the p value adjusted using the false discovery rate. Impact is the pathway impact value calculated from pathway topology analysis.

**Figure 5:**
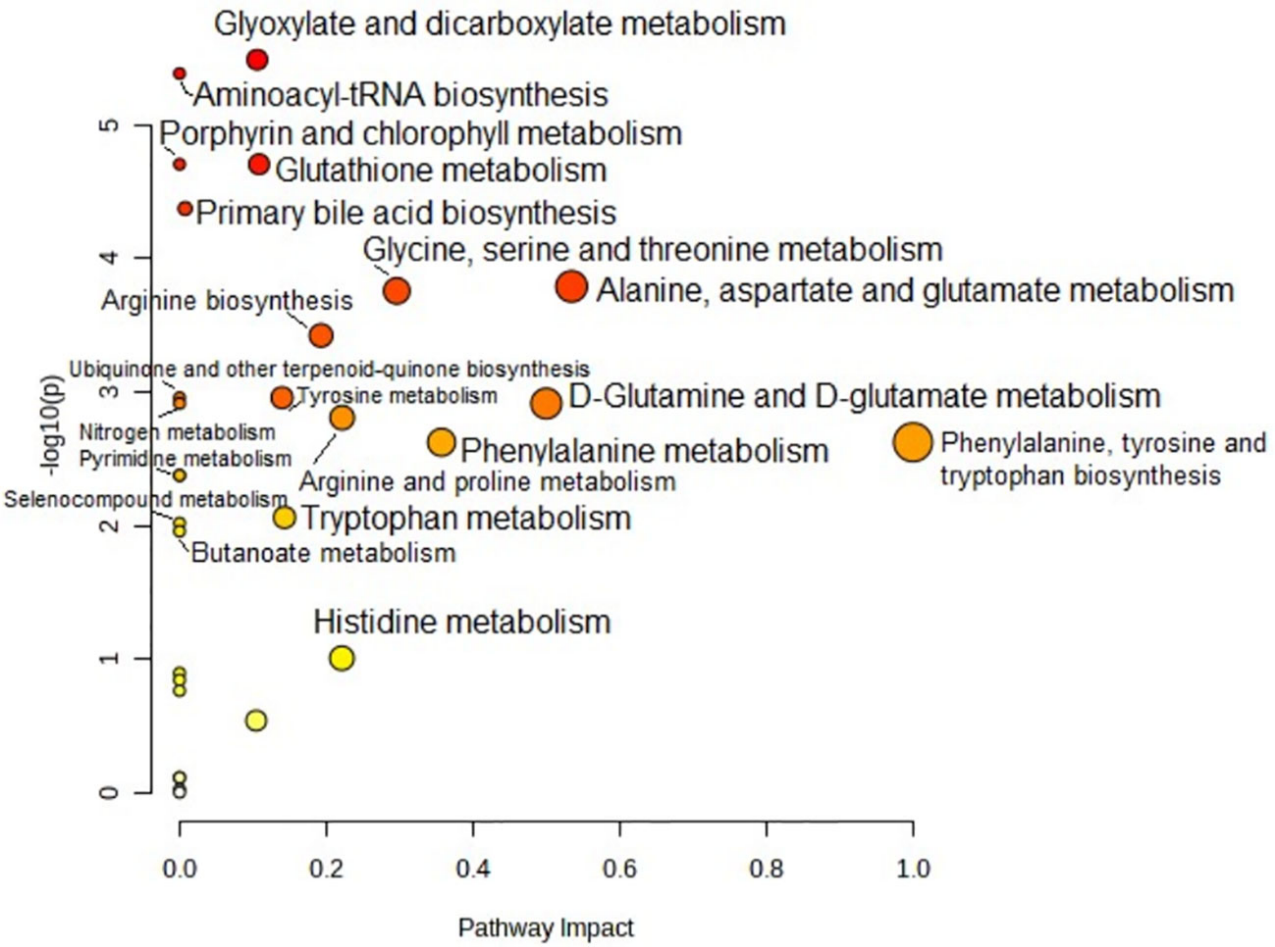
Pathway analysis of serum amino acid profiles of the papillary thyroid carcinoma with Hashimoto group and the control group. Pathway impact values are plotted against the X-axis, and -log (P) values are plotted against the Y-axis. For visual clarification, the pathway importance and the statistical significance are proportional to the node radius and colour, respectively. FDR p is the p value adjusted using the false discovery rate. Impact is the pathway impact value calculated from pathway topology analysis.

Metabolic pathway analysis was performed to interpret the biological relevance of the differences in serum AA profiles in PTC0 and PTC1. The KEGG and HMDB databases were used to analyze twenty-one detected amino acids, and the results were submitted to MetaboAnalyst 5.0 to display the statistical analysis results of informatics analysis. This analysis generates a pathway impact score and the associated p value. A value >0.1 was chosen as the cut-off for less important pathways (**Figures 4**, **5**). All the identified pathways are shown in **Supplementary Tables S2** and **S3**.

Pathway analysis showed that “glyoxylate and dicarboxylate metabolism” was the most significant pathway characteristic of PTC0 (**Figure 4**), which was selected on the basis of disturbed concentrations of glycine, glutamine and glutamic acid in the serum of PTC0 patients compared with healthy controls. However, the pathway “D-Glutamine and D-glutamate metabolism” had the highest FDR value and pathway impact value calculated from pathway topology analysis (**Table S3**). The next most significantly changed pathways were “arginine biosynthesis” and “nitrogen metabolism” (**Table S3**). The most commonly changed AAs in these pathways were glutamine and glutamic acid.

A very similar set of metabolic pathways was observed in the pathway analysis based on changes in the AA profile in patients with PTC1 (**Figure 5**); however, there were higher FDR values and different pathway impact scores (**Table S3**). The two PTC groups were differentiated by the pathway “glutathione metabolism”.

## 4 Discussion

The standard diagnostic tools for PTC are ultrasound and fine needle aspiration biopsies (6). In turn, HT identification is based on clinical symptoms of hypothyroidism, the presence of TPOAbs, and ultrasound features, although seronegative HT can be observed in more than 10% of cases. In such cases, diagnosis is made based on final histopathology. Additional diagnostic tools that may help to identify PTC and distinguish Hashimoto concomitant with PTC may have crucial clinical implications. Indeed, recent guidelines allow less aggressive treatment for PTC in some circumstances, which might significantly reduce postoperative complications (40). However, there is no gold standard that would allow us to distinguish between Hashimoto’s and cancer, and this distinction is of great importance in further management/treatment. In the development of cancer, AA metabolism is reprogrammed. Additionally, HT affects patient catabolism, and preliminary research suggests that increased serum TSH concentration and autoimmune thyroid inflammation are involved in thyroid tumor growth (18). Therefore, is it possible to find the difference between these two diseases based on the amino acid profiles?

To the best of our knowledge, this is the first study to determine AA profiles in serum samples from PTC0 patients and PTC1 patients compared to those of healthy controls.

Lysine was one of the AAs that was elevated in PTC1 compared to PTC0. Lysine affects the production of proteins in muscles and bones, and lysine deficiency causes chronic fatigue, irritability, hair loss, anemia, susceptibility to infection, recurrent herpes and metabolic disorders. Jiang et al. (41) studied the serum of HT patients and showed that lysine degradation pathways had an impact on different clinical stages of HT (41). Additionally, lysine was increased in the serum of HT and Graves’ disease patients (17). In our study, lysine was increased only in the PTC1 group. According to the referenced authors, alterations in lysine degradation affect the occurrence of HT (42).

Other AAs increased in the serum of PTC1 patients compared to HCs were leucine and arginine (**Table S1**). Interestingly, in PTC0 patients, we also observed a strong increase in arginine concentration (**Table S1**). Serum concentrations of arginine were altered in both PTC0 and PTC1 patients, which was also indicated by the MetPA analysis pathway “arginine biosynthesis” (**Figures 4**, **5**). A positive correlation of arginine with fT4 was observed in PTC0 serum (**Table 2**). Interestingly, Ittermann et al. (43) found that in patients with hyperthyroidism, serum concentrations of arginine and its metabolites, including asymmetric and symmetric dimethylarginines and homoarginine, were associated with serum TSH, fT3, and fT4 concentrations. Treatment of hyperthyroidism by antithyroid drugs increased arginine levels (44). In turn, Gluvic et al. (44) described many cases of increased NO bioavailability by levothyroxine therapy. Thyroid hormones stimulate L-arginine uptake by endothelial cells by upregulating L-Arg transporters (45), and arginine is a major regulator of mitochondrial activities in cancer metabolism (31). Supplementation with arginine rewires T-cell metabolism from glycolysis to oxidative phosphorylation and promotes its survival and antitumor ability (31). Lu et al. (46), in H^1^ NMR analysis of plasma from papillary thyroid microcarcinoma patients, reported reduced levels of valine, lysine and leucine compared with healthy groups. In a study by Jiang et al. (41), valine, leucine, and isoleucine degradation and valine, leucine, and isoleucine biosynthesis differentiated euthyroid HT patients from HT patients with subclinical hypothyroidism. However, it should be stressed that Hashimoto disease may influence the metabolism of many other tissues, and this may affect serum AA concentrations. Indeed, we observed elevated concentrations of leucine in serum from HT patients. According to Krishnamurthy et al. (47), arginine, valine, and leucine are important in immunological responses, including the synthesis of various antibodies and the activation of T cells and macrophages. It appears that the deficiency of any essential AAs, including valine, impairs T4 production and leads to primary hypothyroidism (48). Thyroid hormones have a catabolic effect on protein metabolism. In most catabolic states, uptake of branched-chain amino acids from body proteins is reduced; therefore, the increase in their concentrations does not depend on the increase in their content in the diet but results from both their reduced peripheral metabolism and increased release from fat-free tissues (49). Additionally, in our study, the concentration of valine was elevated in both PTC groups; however, much higher differences in valine levels were observed in PTC1 patients. Plasma branched-chain amino acids are decreased in short-term profound hypothyroidism and increase in response to thyroid hormone supplementation (16). Therefore, thyroid hormone supplementation can be a reason for the higher serum concentrations of BCAAs in PTC1 patients (**Table S1**). The next amino acid, glycine is a highly desirable compound for cancer cells (24, 33)., therefore, reduced levels of glycine in the serum of PTC patients (**Table S1**) could be the reason for glycine participation in cancer pathogenesis (21). We observed an inverse correlation of glycine with fT4 in PTC0 serum (**Table 2**). Glycine supplementation improves the conversion of fT4 to fT3, which contributes to the proper functioning of the thyroid gland. Mannisto et al. reported that intraperitoneal administration of glycine inhibited TSH secretion in rats (50).

In turn, glutamate and aspartate, which are excitatory amino acids, act by increasing the concentrations of TSH, fT3 and fT4 in rat serum (51). Indeed, in our study, glutamate and aspartate were positively correlated with TSH in the serum of PTC1 patients. Moreover, significantly higher concentrations of glutamate and aspartic acid were detected in the serum of PTC0 and PTC1 patients in comparison to healthy controls (**Table S1**). Aizawa et al. (52) studied the effects of glutamic acid and glutamine on TSHβ expression in pars tuberalis (PT) slice cultures from rat brains. After 2- and 4-h treatments, glutamic acid and glutamine significantly stimulated TSHβ expression in PT slices, and the impact of glutamic acid was stronger than that of glutamine (52). TSH was also positively correlated with aspartic acid, although the correlation was weak (0.356, p<0.05) (**Table 3**). The enzymes involved in glutaminolysis were overexpressed in thyroid cancer tissue (20, 21, 53) and promoted the transformation of glutamine to glutamate to sustain the TCA cycle and anabolic processes (27). Therefore, excess products of glutaminolysis, such as aspartic and glutamic acid, can be removed into the serum of PTC patients. Furthermore, aspartic and glutamic acid are substrates for nucleotide biosynthesis, and increased amounts could replenish the levels of the metabolites of the TCA cycle that may be decreased as a result of aerobic glycolysis (Warburg effect) (25). According to Cheng et al. (54), the increased glutamate concentrations in PTC patients are a result of increased glutamine metabolism in tumour cells. Moreover, the existing association of thyroid autoimmunity with PTC may be involved in increased serum glutamine concentrations (18). One of the amino acids with a reduced concentration in serum samples in both the PTC0 and PTC1 groups compared to HCs was alanine. Additionally, Qing Huang et al. (25) found reduced concentrations of alanine in the serum of PTC patients. A similar reason is indicated by Wojtowicz et al. (26). Decreased alanine might be evidence of its fast utilization from circulating blood as an answer for energy demands (26). In our study, alanine showed a decreasing trend in the serum of PTC1 patients compared to PTC0 patients. Additionally, we observed a strong negative correlation of alanine with TPO-Abs in the serum of PTC1 patients. Thyroid hormones control a multitude of homeostatic functions, including protein proteolysis (16). In Hashimoto, the antibody titre is significantly elevated, so this may be another factor lowering the concentration of alanine in patients with HT. When comparing different studies, the differences in the abundance of these amino acids between healthy subjects and patients with benign or malignant thyroid lesions do not always match. Certainly, observed results need further investigations. One of this reason might be the use of different sampling methods, techniques and study groups. However, there is agreement that plasma/serum levels of tyrosine, a precursor of thyroid hormones, are lower in PTC patients than in controls (26, 46, 55–57). The T3 hormone, triiodothyronine, constitutes only 10% of the total thyroid hormones, although it is considered responsible for most of the thyroid’s activities and is 3-4 times stronger than the T4 hormone. Tyrosine is necessary for synthesis of thyroxine, which is produced by the thyroid gland (47). Reduced values of fT4 and elevated values of fT3 accompany Hashimoto (18). Deficiency in tyrosine, as well as phenylalanine, results in altered levels of thyroid hormones (47). Tyrosine is considered a nonessential amino acid because it can be synthesized from phenylalanine; nonetheless, it has an important role in the production of proteins that are a part of signal transduction processes, acting as a receiver of phosphate groups transferred through tyrosine kinases. In turn, these enzymes have been associated with the regulation of cellular proliferation, survival, differentiation, function and motility, linking them to a cancer phenotype Tahara et al. (48) demonstrated the effects of amino acid deficiency on serum levels of T4, T3, fT4, and reverse T3; they reported that reduction of phenylalanine and tyrosine drastically affected the serum levels of thyroid hormones. We observed a positive correlation between tyrosine and fT3 and fT4 in the serum of PTC1 patients. Interestingly, in HT and other autoimmune thyroid diseases, other studies (17, 18) did not observe the phenomenon of decreased levels of tyrosine in serum patients. Jiang et al. (41) suggested that lysine degradation and tyrosine metabolism played an important role in the HTS group compared to the control group. However, this was not supported by measured tyrosine concentrations, only enrichment analysis (41). This may be due to the effects of the HT drugs.

### 4.1 Conclusion

Our study aimed to contribute to further understanding of how AAs differ between patients with papillary thyroid cancer alone and those with comorbid Hashimoto thyroiditis in relation to healthy controls. By examining the amino acid profile in the blood, we found some unique patterns that would allow us to distinguish PTC0 patients from PTC1 patients. The clinical significance of these findings remains unclear. This is due to several limitations (1), absence of serum from the HT group, (2) relatively small study groups, (3) absence of laboratory tests of thyroid function or thyroid autoantibodies from control group. Therefore, despite finding differences in several AAs depending on the analysis used, only two were actually changed in most analyses and could be used to distinguish the studied PTC groups. The AA that most strongly separated PTC0 from PTC1 was lysine. Lysine, in addition to glutamic acid, differentiated both PTC groups and was positively correlated with anti-TG. The second AA marker with high probability may be alanine. Although no statistically significant difference was found (probably due to high SD), its concentration showed a downwards trend in the PTC1 group compared to the PTC0 and HC groups. Alanine was also negatively correlated with anti-TPO and was one of the 8 markers of AAs that separated/distinguished PTC1 patients from healthy subjects based on ROC analysis. We believe that long-term studies in larger populations are needed to confirm the predictive potential of selected metabolites in diagnosing thyroid lesions.

## Data availability statement

The data presented in the study are deposited in the MetaboLights repository, accession number MTBLS8012; https://www.ebi.ac.uk/metabolights/editor/study/MTBLS8012.

## Ethics statement

The studies involving human participants were reviewed and approved by Independent Bioethics Committee for Scientific Research at the Medical University of Gdansk under number NKBBN/62/2021. Written informed consent to participate in this study was provided by the participants’ legal guardian/next of kin.

## Author contributions

AH and AM, designed the study, analyzed and interpreted results, and wrote the manuscript. AH, and AT, contributed to sample and clinical data collection. JT, conducted the amino acids analysis. JK conducted the analysis of mRNA levels by real-time PCR. AZ and AM, performed the statistical analysis. All authors reviewed and edited the manuscript.
