# Supplementary material for: Alterations in the amino acid profile in patients with papillary thyroid carcinoma with and without Hashimoto’s thyroiditis
Source: Front Endocrinol (Lausanne). 2023 Aug 18;14:1199291. doi: 10.3389/fendo.2023.1199291 (PMC10471980; doi:10.3389/fendo.2023.1199291)
Supplement: Supplementary file 1 [file Table_1.doc]

**Alterations in the amino acid profile in patients with papillary thyroid carcinoma with and without Hashimoto’s thyroiditis**

**Table S1a.** Average values ​​of amino acids concentrations (µM/L) in in healthy control (HC), patients with PTC without Hashimoto thyroiditis (PTC0) and patients with Hashimoto thyroiditis (PTC1). Values are mean ± SD. NS – not significant.

|  | HC | PTC0 | PTC1 | HC vs PTC 0 | HC vs PTC 1 | PTC 0 vs PTC1 |
| --- | --- | --- | --- | --- | --- | --- |
| Aspartic acid | 20.9 ± 8.99 | 29.9 ± 12.6 | 29.4 ± 12.3 | 0.046* | 0.026* | NS |
| Asparagine | 39.6 ± 11.8 | 41.4 ± 9.42 | 36.7 ± 10.5 | NS | NS | NS |
| Glycine | 356 ± 140 | 244 ± 112 | 251 ± 89.3 | 0.008* | 0.027* | NS |
| Glutamine | 365 ± 129 | 486 ± 91.6 | 518 ± 96.3 | 0.002* | < 0.001* | NS |
| Glutamic acid | 60.2 ± 15.0 | 86.4 ± 29.5 | 141 ± 67.8 | 0.026* | < 0.001* | 0.003* |
| Serine | 259 ± 95.1 | 268 ± 94.2 | 233 ± 69.7 | NS | NS | NS |
| Betaine | 33.8 ± 8.46 | 29.7 ± 5.99 | 31.8 ± 10.9 | NS | NS | NS |
| Threonine | 123 ± 33.4 | 118 ± 32.7 | 120 ± 31.6 | NS | NS | NS |
| Alanine | 942 ± 200 | 806 ± 199 | 764 ± 224 | 0.044 | 0.004 | NS |
| Proline | 172 ± 42.5 | 169 ± 53.8 | 186 ± 81.5 | NS | NS | NS |
| Creatinine | 75.3 ± 11.7 | 77.9 ± 12.6 | 82.4 ± 17.0 | NS | NS | NS |
| Valine | 149 ± 24.7 | 177 ± 42.5 | 186 ± 44.9 | 0.037* | < 0.001* | NS |
| Methionine | 61.2 ± 17.4 | 55.7 ± 13.5 | 56.0 ± 10.4 | NS | NS | NS |
| Tyrosine | 60.3 ± 16.1 | 44.6 ± 14.2 | 45.5 ± 12.5 | < 0.001 | < 0.001 | NS |
| Histidine | 69.7 ± 11.3 | 70.7 ± 14.2 | 79.8 ± 13.3 | NS | 0.043* | NS |
| Isoleucine | 57.3 ± 15.0 | 55.3 ± 12.3 | 56.0 ± 15.5 | NS | NS | NS |
| Lysine | 55.3 ± 20.9 | 50.0 ± 24.5 | 71.5 ± 28.7 | NS | NS | 0.013* |
| Leucine | 105 ± 17.5 | 112 ± 24.1 | 127 ± 33.0 | NS | 0.019* | NS |
| Arginine | 74.3 ± 27.2 | 92.1 ± 18.7 | 95.6 ± 15.9 | NS | 0.014* | NS |
| Phenylalanine | 87.5 ± 11.6 | 94.4 ± 21.0 | 95.0 ± 17.9 | NS | NS | NS |
| Tryptophan | 48.8 ± 10.4 | 45.3 ± 8.44 | 45.0 ± 8.74 | NS | NS | NS |

p from One Way Analysis of Variance followed by All Pairwise Comparison Holm-Sidak Method, * p from non-parametric Kruskall-Wallis One Way Analysis of Variance followed by All Pairwise Comparison Dunn’s Method on ranks.

**Table S1b.** Statistical parameters for non-parametric Kruskall-Wallis One Way Analysis of Variance followed by All Pairwise Comparison Dunn’s Method on ranks.

|  | ANOVA | | | | | All Pairwise Comparison Dunn’s Method on ranks | | | |
| --- | --- | --- | --- | --- | --- | --- | --- | --- | --- |
|  |  | Median | 75% | 25% | p |  | Diff of ranks | Q | p |
| Aspartic acid | HC | 18.5 | 13.25 | 27.50 | **0.013** | PTC0 vs HC | 15.174 | 2.426 | 0.046 |
|  | PTC0 | 25.0 | 20.75 | 40.75 |  | PTC0 vs PTC1 | 0.426 | 0.0698 | 1.000 |
|  | PTC1 | 24.0 | 20.00 | 40.00 |  | PTC1 vs HC | 14.748 | 2.620 | 0.026 |
| Asparagine | HC | 40.0 | 29.00 | 46.00 | 0.175 | - | - | - | - |
|  | PTC0 | 40.0 | 33.00 | 50.00 |  | - | - | - | - |
|  | PTC1 | 36.0 | 28.25 | 45.75 |  | - | - | - | - |
| Glycine | HC | 319 | 246.5 | 471.5 | **0.011** | HC vs PTC0 | 19.790 | 2.980 | 0.009 |
|  | PTC0 | 235 | 149.5 | 328.5 |  | HC vs PTC1 | 11.879 | 1.902 | 0.172 |
|  | PTC1 | 262 | 188.0 | 360.0 |  | PTC1 vs PTC0 | 7.911 | 1.252 | 0.632 |
| Glutamine | HC | 336.5 | 266.5 | 420.5 | **˂0.001** | PTC1 vs HC | 30.002 | 4.488 | <0.001 |
|  | PTC0 | 486.0 | 403.0 | 551.0 |  | PTC1 vs PTC0 | 5.794 | 0.876 | 1.000 |
|  | PTC1 | 511.0 | 460.5 | 563.5 |  | PTC0 vs HC | 24.207 | 3.372 | 0.002 |
| Glutamic acid | HC | 59.0 | 48.5 | 67.5 | **˂0.001** | PTC1 vs HC | 35.648 | 5.854 | <0.001 |
|  | PTC0 | 85.5 | 58.3 | 111.8 |  | PTC1 vs PTC0 | 19.252 | 3.279 | 0.003 |
|  | PTC1 | 121.0 | 97.0 | 163.0 |  | PTC0 vs HC | 16.396 | 2.622 | 0.026 |
| Serine | HC | 262.5 | 162.0 | 334.0 | 0.444 | - | - | - | - |
|  | PTC0 | 261.0 | 168.5 | 360.0 |  | - | - | - | - |
|  | PTC1 | 215.0 | 183.0 | 283.0 |  | - | - | - | - |
| Betaine | HC | 33.0 | 27.0 | 40.0 | 0.251 | - | - | - | - |
|  | PTC0 | 30.0 | 26.0 | 33.0 |  | - | - | - | - |
|  | PTC1 | 29.0 | 23.0 | 38.5 |  | - | - | - | - |
| Threonine | HC | 122.0 | 96.3 | 136.0 | 0.903 | - | - | - | - |
|  | PTC0 | 109.0 | 91.0 | 145.0 |  | - | - | - | - |
|  | PTC1 | 118.5 | 91.0 | 143.8 |  | - | - | - | - |
| Proline | HC | 170.0 | 150.0 | 186.0 | 0.667 | - | - | - | - |
|  | PTC0 | 151.5 | 126.3 | 196.5 |  | - | - | - | - |
|  | PTC1 | 162.5 | 135.03 | 202.8 |  | - | - | - | - |
| Valine | HC | 148.0 | 125.53 | 166.5 | **0.001** | PTC1 vs HC | 24.250 | 3.666 | <0.001 |
|  | PTC0 | 169.5 | 139.8 | 199.3 |  | PTC1 vs PTC0 | 6.366 | 0.974 | 0.990 |
|  | PTC1 | 180.0 | 149.5 | 201.0 |  | PTC0 vs HC | 17.884 | 2.499 | 0.037 |
| Histidine | HC | 70.0 | 63.0 | 80.0 | **0.023** | PTC1 vs HC | 15.911 | 2.446 | 0.043 |
|  | PTC0 | 72.0 | 58.8 | 82.5 |  | PTC1 vs PTC0 | 14.199 | 2.183 | 0.087 |
|  | PTC1 | 78.0 | 68.3 | 91.3 |  | PTC0 vs HC | 1.712 | 0.247 | 1.000 |
| Isoleucine | HC | 57.0 | 48.0 | 70.0 | 0.677 | - | - | - | - |
|  | PTC0 | 53.0 | 47.0 | 63.0 |  | - | - | - | - |
|  | PTC1 | 50.5 | 44.0 | 63.8 |  | - | - | - | - |
| Lysine | HC | 58.0 | 37.0 | 69.0 | **0.014** | PTC1 vs PTC0 | 16.748 | 2.860 | 0.013 |
|  | PTC0 | 43.0 | 29.0 | 61.0 |  | PTC1 vs HC | 10.646 | 1.773 | 0.229 |
|  | PTC1 | 66.0 | 47.0 | 94.0 |  | HC vs PTC0 | 6.102 | 0.980 | 0.982 |
| Leucine | HC | 106.0 | 90.8 | 118.8 | **0.022** | PTC1 vs HC | 17.744 | 2.734 | 0.019 |
|  | PTC0 | 113.5 | 96.0 | 131.3 |  | PTC1 vs PTC0 | 10.449 | 1.578 | 0.344 |
|  | PTC1 | 116.0 | 101.5 | 140.0 |  | PTC0 vs HC | 7.295 | 1.060 | 0.867 |
| Arginine | HC | 81.0 | 48.0 | 101.0 | **0.016** | PTC1 vs HC | 18.963 | 2.827 | 0.014 |
|  | PTC0 | 88.0 | 81.0 | 108.0 |  | PTC1 vs PTC0 | 4.181 | 0.654 | 1.000 |
|  | PTC1 | 98.0 | 83.5 | 107.5 |  | PTC0 vs HC | 14.782 | 2.062 | 0.118 |
| Phenylalanine | HC | 87.0 | 80.3 | 95.3 | 0.367 | - | - | - | - |
|  | PTC0 | 87.0 | 79.0 | 103.5 |  | - | - | - | - |
|  | PTC1 | 94.0 | 80.3 | 104.8 |  | - | - | - | - |
| Tryptophan | HC | 46.0 | 40.3 | 58.5 | 0.368 | - | - | - | - |
|  | PTC0 | 43.0 | 38.8 | 52.0 |  | - | - | - | - |
|  | PTC1 | 43.5 | 37.0 | 52.3 |  | - | - | - | - |

**Table S1c.** Statistical parameters for One Way Analysis of Variance followed by All Pairwise Comparison Holm-Sidak Method.

|  | ANOVA | | | | | | | All Pairwise Comparison Holm-Sidak Method. | | | |
| --- | --- | --- | --- | --- | --- | --- | --- | --- | --- | --- | --- |
|  |  | Mean ± SD | DF | SS | MS | F | p |  | Diff of ranks | Q | p |
| Alanine | HC | 942 ± 200 | 2 | 505912 | 252956 | 5.542 | **0.005** | HC vs PTC1 | 177.13 | 3.275 | 0.004 |
|  | PTC0 | 806 ± 199 |  |  |  |  |  | HC vs PTC0 | 135.3 | 2.325 | 0.044 |
|  | PTC1 | 764 ± 224 |  |  |  |  |  | PTC0 vs PTC1 | 41.843 | 0.791 | 0.431 |
| Creatinine | HC | 75.3 ± 11.7 | 2 | 826.9 | 413.4 | 1.952 | 0.148 | - | - | - | - |
|  | PTC0 | 77.9 ± 12.6 |  |  |  |  |  | - | - | - | - |
|  | PTC1 | 82.4 ± 17.0 |  |  |  |  |  | - | - | - | - |
| Methionine | HC | 61.2 ± 17.4 | 2 | 523.4 | 261.7 | 1.371 | 0.259 | - | - | - | - |
|  | PTC0 | 55.7 ± 13.5 |  |  |  |  |  | - | - | - | - |
|  | PTC1 | 56.0 ± 10.4 |  |  |  |  |  | - | - | - | - |
| Tyrosine | HC | 60.3 ± 16.1 | 2 | 4101 | 2050 | 9.947 | **<0.001** | HC vs PTC1 | 14.77 | 3.927 | <0.001 |
|  | PTC0 | 44.6 ± 14.2 |  |  |  |  |  | HC vs PTC0 | 15.657 | 3.927 | <0.001 |
|  | PTC1 | 45.5 ± 12.5 |  |  |  |  |  | PTC1 v. PTC0 | 0.885 | 0.241 | 0.810 |

**Table S2**. Result from Pathway Analysis of PTC0 against HC.

|  | **Match status^1^** | **p^2^** | **Holm p^3^** | **FDR^4^** | **Impact^5^** |
| --- | --- | --- | --- | --- | --- |
| Glyoxylate and dicarboxylate metabolism | [3/32](https://www.metaboanalyst.ca/MetaboAnalyst/Secure/pathway/PathResultView.xhtml) | 1.5948E-5 | 4.6248E-4 | 3.3195E-4 | 0.10582 |
| Arginine biosynthesis | [4/14](https://www.metaboanalyst.ca/MetaboAnalyst/Secure/pathway/PathResultView.xhtml) | 2.2893E-5 | 6.4101E-4 | 3.3195E-4 | 0.19289 |
| D-Glutamine and D-glutamate metabolism | [2/6](https://www.metaboanalyst.ca/MetaboAnalyst/Secure/pathway/PathResultView.xhtml) | 4.8735E-5 | 0.0013158 | 3.5333E-4 | 0.5 |
| Nitrogen metabolism | [2/6](https://www.metaboanalyst.ca/MetaboAnalyst/Secure/pathway/PathResultView.xhtml) | 4.8735E-5 | 0.0013158 | 3.5333E-4 | 0.0 |
| Arginine and proline metabolism | [3/38](https://www.metaboanalyst.ca/MetaboAnalyst/Secure/pathway/PathResultView.xhtml) | 3.4871E-4 | 0.0087178 | 0.0020225 | 0.22166 |
| Aminoacyl-tRNA biosynthesis | [18/48](https://www.metaboanalyst.ca/MetaboAnalyst/Secure/pathway/PathResultView.xhtml) | 5.339E-4 | 0.012814 | 0.0025109 | 0.0 |
| Glutathione metabolism | [2/28](https://www.metaboanalyst.ca/MetaboAnalyst/Secure/pathway/PathResultView.xhtml) | 6.9268E-4 | 0.015932 | 0.0025109 | 0.10839 |
| Porphyrin and chlorophyll metabolism | [2/30](https://www.metaboanalyst.ca/MetaboAnalyst/Secure/pathway/PathResultView.xhtml) | 6.9268E-4 | 0.015932 | 0.0025109 | 0.0 |
| Alanine, aspartate and glutamate metabolism | [5/28](https://www.metaboanalyst.ca/MetaboAnalyst/Secure/pathway/PathResultView.xhtml) | 8.7408E-4 | 0.018356 | 0.0028165 | 0.53446 |
| Purine metabolism | [1/65](https://www.metaboanalyst.ca/MetaboAnalyst/Secure/pathway/PathResultView.xhtml) | 0.001164 | 0.02328 | 0.0030687 | 0.0 |
| Pyrimidine metabolism | [1/39](https://www.metaboanalyst.ca/MetaboAnalyst/Secure/pathway/PathResultView.xhtml) | 0.001164 | 0.02328 | 0.0030687 | 0.0 |
| Butanoate metabolism | [1/15](https://www.metaboanalyst.ca/MetaboAnalyst/Secure/pathway/PathResultView.xhtml) | 0.0029617 | 0.053311 | 0.0071575 | 0.0 |
| Primary bile acid biosynthesis | [1/46](https://www.metaboanalyst.ca/MetaboAnalyst/Secure/pathway/PathResultView.xhtml) | 0.010855 | 0.18454 | 0.024215 | 0.00758 |
| Glycine, serine and threonine metabolism | [3/33](https://www.metaboanalyst.ca/MetaboAnalyst/Secure/pathway/PathResultView.xhtml) | 0.029407 | 0.47051 | 0.060915 | 0.29611 |
| Histidine metabolism | [3/16](https://www.metaboanalyst.ca/MetaboAnalyst/Secure/pathway/PathResultView.xhtml) | 0.032201 | 0.48302 | 0.062256 | 0.22131 |
| Tyrosine metabolism | [1/42](https://www.metaboanalyst.ca/MetaboAnalyst/Secure/pathway/PathResultView.xhtml) | 0.044842 | 0.62779 | 0.076495 | 0.13972 |
| Ubiquinone and other terpenoid-quinone biosynthesis | [1/9](https://www.metaboanalyst.ca/MetaboAnalyst/Secure/pathway/PathResultView.xhtml) | 0.044842 | 0.62779 | 0.076495 | 0.0 |
| Tryptophan metabolism | [1/41](https://www.metaboanalyst.ca/MetaboAnalyst/Secure/pathway/PathResultView.xhtml) | 0.054959 | 0.65951 | 0.088545 | 0.14305 |
| Pantothenate and CoA biosynthesis | [2/19](https://www.metaboanalyst.ca/MetaboAnalyst/Secure/pathway/PathResultView.xhtml) | 0.093349 | 1.0 | 0.14248 | 0.0 |
| Phenylalanine, tyrosine and tryptophan biosynthesis | [2/4](https://www.metaboanalyst.ca/MetaboAnalyst/Secure/pathway/PathResultView.xhtml) | 0.13385 | 1.0 | 0.18485 | 1.0 |
| Phenylalanine metabolism | [2/10](https://www.metaboanalyst.ca/MetaboAnalyst/Secure/pathway/PathResultView.xhtml) | 0.13385 | 1.0 | 0.18485 | 0.35714 |
| Valine, leucine and isoleucine degradation | [3/40](https://www.metaboanalyst.ca/MetaboAnalyst/Secure/pathway/PathResultView.xhtml) | 0.19573 | 1.0 | 0.25801 | 0.0 |
| Selenocompound metabolism | [1/20](https://www.metaboanalyst.ca/MetaboAnalyst/Secure/pathway/PathResultView.xhtml) | 0.27527 | 1.0 | 0.34708 | 0.0 |
| Valine, leucine and isoleucine biosynthesis | [4/8](https://www.metaboanalyst.ca/MetaboAnalyst/Secure/pathway/PathResultView.xhtml) | 0.31282 | 1.0 | 0.37799 | 0.0 |
| Cysteine and methionine metabolism | [1/33](https://www.metaboanalyst.ca/MetaboAnalyst/Secure/pathway/PathResultView.xhtml) | 0.3661 | 1.0 | 0.42468 | 0.10446 |
| Nicotinate and nicotinamide metabolism | [1/15](https://www.metaboanalyst.ca/MetaboAnalyst/Secure/pathway/PathResultView.xhtml) | 0.66449 | 1.0 | 0.74117 | 0.0 |
| beta-Alanine metabolism | [2/21](https://www.metaboanalyst.ca/MetaboAnalyst/Secure/pathway/PathResultView.xhtml) | 0.89695 | 1.0 | 0.93851 | 0.0 |
| Lysine degradation | [1/25](https://www.metaboanalyst.ca/MetaboAnalyst/Secure/pathway/PathResultView.xhtml) | 0.93851 | 1.0 | 0.93851 | 0.0 |
| Biotin metabolism | [1/10](https://www.metaboanalyst.ca/MetaboAnalyst/Secure/pathway/PathResultView.xhtml) | 0.93851 | 1.0 | 0.93851 | 0.0 |

^1^Match status: actually, matched number from the data among total number of compounds in the pathway; ^2^Raw p: original p value calculated from the enrichment analysis; ^3^Holm p: p value adjusted by Holm-Bonferroni method; ^4^FDR: p value adjusted using False Discovery Rate; ^5^Impact is the pathway impact value calculated from pathway topology analysis. HC – healthy control, PTC0- papillary thyroid cancer subjects without hashimoto thyroiditis.

**Table S3**. Result from Pathway Analysis of PTC1 against HC.

|  | **Match status^1^** | **p^2^** | **Holm p^3^** | **FDR^4^** | **Impact^5^** |
| --- | --- | --- | --- | --- | --- |
| Glyoxylate and dicarboxylate metabolism | [3/32](https://www.metaboanalyst.ca/MetaboAnalyst/Secure/pathway/PathResultView.xhtml) | 3.2532E-6 | 9.4341E-5 | 5.9971E-5 | 0.10582 |
| Aminoacyl-tRNA biosynthesis | [18/48](https://www.metaboanalyst.ca/MetaboAnalyst/Secure/pathway/PathResultView.xhtml) | 4.1359E-6 | 1.1581E-4 | 5.9971E-5 | 0.0 |
| Glutathione metabolism | [2/28](https://www.metaboanalyst.ca/MetaboAnalyst/Secure/pathway/PathResultView.xhtml) | 1.9754E-5 | 5.3335E-4 | 1.4322E-4 | 0.10839 |
| Porphyrin and chlorophyll metabolism | [2/30](https://www.metaboanalyst.ca/MetaboAnalyst/Secure/pathway/PathResultView.xhtml) | 1.9754E-5 | 5.3335E-4 | 1.4322E-4 | 0.0 |
| Primary bile acid biosynthesis | [1/46](https://www.metaboanalyst.ca/MetaboAnalyst/Secure/pathway/PathResultView.xhtml) | 4.2382E-5 | 0.0010595 | 2.4581E-4 | 0.00758 |
| Alanine, aspartate and glutamate metabolism | [5/28](https://www.metaboanalyst.ca/MetaboAnalyst/Secure/pathway/PathResultView.xhtml) | 1.6214E-4 | 0.0038912 | 7.2583E-4 | 0.53446 |
| Glycine, serine and threonine metabolism | [3/33](https://www.metaboanalyst.ca/MetaboAnalyst/Secure/pathway/PathResultView.xhtml) | 1.752E-4 | 0.0040296 | 7.2583E-4 | 0.29611 |
| Arginine biosynthesis | [4/14](https://www.metaboanalyst.ca/MetaboAnalyst/Secure/pathway/PathResultView.xhtml) | 3.7788E-4 | 0.0083134 | 0.0013698 | 0.19289 |
| Tyrosine metabolism | [1/42](https://www.metaboanalyst.ca/MetaboAnalyst/Secure/pathway/PathResultView.xhtml) | 0.0011052 | 0.023209 | 0.002954 | 0.13972 |
| Ubiquinone and other terpenoid-quinone biosynthesis | [1/9](https://www.metaboanalyst.ca/MetaboAnalyst/Secure/pathway/PathResultView.xhtml) | 0.0011052 | 0.023209 | 0.002954 | 0.0 |
| D-Glutamine and D-glutamate metabolism | [2/6](https://www.metaboanalyst.ca/MetaboAnalyst/Secure/pathway/PathResultView.xhtml) | 0.0012223 | 0.023224 | 0.002954 | 0.5 |
| Nitrogen metabolism | [2/6](https://www.metaboanalyst.ca/MetaboAnalyst/Secure/pathway/PathResultView.xhtml) | 0.0012223 | 0.023224 | 0.002954 | 0.0 |
| Arginine and proline metabolism | [3/38](https://www.metaboanalyst.ca/MetaboAnalyst/Secure/pathway/PathResultView.xhtml) | 0.0015061 | 0.025605 | 0.0033599 | 0.22166 |
| Phenylalanine, tyrosine and tryptophan biosynthesis | [2/4](https://www.metaboanalyst.ca/MetaboAnalyst/Secure/pathway/PathResultView.xhtml) | 0.0023825 | 0.038119 | 0.0046061 | 1.0 |
| Phenylalanine metabolism | [2/10](https://www.metaboanalyst.ca/MetaboAnalyst/Secure/pathway/PathResultView.xhtml) | 0.0023825 | 0.038119 | 0.0046061 | 0.35714 |
| Purine metabolism | [1/65](https://www.metaboanalyst.ca/MetaboAnalyst/Secure/pathway/PathResultView.xhtml) | 0.004218 | 0.059053 | 0.0071955 | 0.0 |
| Pyrimidine metabolism | [1/39](https://www.metaboanalyst.ca/MetaboAnalyst/Secure/pathway/PathResultView.xhtml) | 0.004218 | 0.059053 | 0.0071955 | 0.0 |
| Tryptophan metabolism | [1/41](https://www.metaboanalyst.ca/MetaboAnalyst/Secure/pathway/PathResultView.xhtml) | 0.008708 | 0.1045 | 0.01403 | 0.14305 |
| Selenocompound metabolism | [1/20](https://www.metaboanalyst.ca/MetaboAnalyst/Secure/pathway/PathResultView.xhtml) | 0.0096058 | 0.10566 | 0.014661 | 0.0 |
| Butanoate metabolism | [1/15](https://www.metaboanalyst.ca/MetaboAnalyst/Secure/pathway/PathResultView.xhtml) | 0.011047 | 0.11047 | 0.016019 | 0.0 |
| Histidine metabolism | [3/16](https://www.metaboanalyst.ca/MetaboAnalyst/Secure/pathway/PathResultView.xhtml) | 0.098576 | 0.88718 | 0.13613 | 0.22131 |
| Valine, leucine and isoleucine degradation | [3/40](https://www.metaboanalyst.ca/MetaboAnalyst/Secure/pathway/PathResultView.xhtml) | 0.12744 | 1.0 | 0.16798 | 0.0 |
| Pantothenate and CoA biosynthesis | [2/19](https://www.metaboanalyst.ca/MetaboAnalyst/Secure/pathway/PathResultView.xhtml) | 0.14325 | 1.0 | 0.18062 | 0.0 |
| Valine, leucine and isoleucine biosynthesis | [4/8](https://www.metaboanalyst.ca/MetaboAnalyst/Secure/pathway/PathResultView.xhtml) | 0.17239 | 1.0 | 0.2083 | 0.0 |
| Cysteine and methionine metabolism | [1/33](https://www.metaboanalyst.ca/MetaboAnalyst/Secure/pathway/PathResultView.xhtml) | 0.28872 | 1.0 | 0.33491 | 0.10446 |
| Lysine degradation | [1/25](https://www.metaboanalyst.ca/MetaboAnalyst/Secure/pathway/PathResultView.xhtml) | 0.77159 | 1.0 | 0.82875 | 0.0 |
| Biotin metabolism | [1/10](https://www.metaboanalyst.ca/MetaboAnalyst/Secure/pathway/PathResultView.xhtml) | 0.77159 | 1.0 | 0.82875 | 0.0 |
| Nicotinate and nicotinamide metabolism | [1/15](https://www.metaboanalyst.ca/MetaboAnalyst/Secure/pathway/PathResultView.xhtml) | 0.94593 | 1.0 | 0.97972 | 0.0 |
| beta-Alanine metabolism | [2/21](https://www.metaboanalyst.ca/MetaboAnalyst/Secure/pathway/PathResultView.xhtml) | 0.99617 | 1.0 | 0.99617 | 0.0 |

^1^Match status: actually, matched number from the data among total number of compounds in the pathway; ^2^Raw p: original p value calculated from the enrichment analysis; ^3^Holm p: p value adjusted by Holm-Bonferroni method; ^4^FDR: p value adjusted using False Discovery Rate; ^5^Impact is the pathway impact value calculated from pathway topology analysis. HC – healthy control, PTC1- papillary thyroid cancer subjects with hashimoto thyroiditis.
